# Predictive Analysis of Linoleic Acid in Red Meat Employing Advanced Ensemble Models of Bayesian and CNN-Bi-LSTM Decision Layer Fusion Based Hyperspectral Imaging

**DOI:** 10.3390/foods13030424

**Published:** 2024-01-28

**Authors:** Xiuwei Yan, Sijia Liu, Songlei Wang, Jiarui Cui, Yongrui Wang, Yu Lv, Hui Li, Yingjie Feng, Ruiming Luo, Zhifeng Zhang, Lei Zhang

**Affiliations:** 1 College of Food Science and Engineering, Ningxia University, Yinchuan 750021, China; xiuweiyan193@stu.nxu.edu.cn (X.Y.); 18309330818@163.com (S.L.); 18366756059@163.com (Y.L.); 15709582584@163.com (H.L.); fengyj@gmail.com (Y.F.); zhanglei_2819@163.com (L.Z.); 2 College of Animal Science and Technology, Ningxia University, Yinchuan 750021, China; c15145244344@163.com (J.C.); wyr2013tymk@163.com (Y.W.); ruimingluonxu@163.com (R.L.); 3 College of Aquaculture, Huazhong Agricultural University, Wuhan 430070, China; 252982125@163.com

**Keywords:** visible near-infrared hyperspectral imaging, red meat, linoleic acid, long short-term memory network, Bayes

## Abstract

Rapid non-destructive testing technologies are effectively used to analyze and evaluate the linoleic acid content while processing fresh meat products. In current study, hyperspectral imaging (HSI) technology was combined with deep learning optimization algorithm to model and analyze the linoleic acid content in 252 mixed red meat samples. A comparative study was conducted by experimenting mixed sample data preprocessing methods and feature wavelength extraction methods depending on the distribution of linoleic acid content. Initially, convolutional neural network Bi-directional long short-term memory (CNN-Bi-LSTM) model was constructed to reduce the loss of the fully connected layer extracted feature information and optimize the prediction effect. In addition, the prediction process of overfitting phenomenon in the CNN-Bi-LSTM model was also targeted. The Bayesian-CNN-Bi-LSTM (Bayes-CNN-Bi-LSTM) model was proposed to improve the linoleic acid prediction in red meat through iterative optimization of Gaussian process acceleration function. Results showed that best preprocessing effect was achieved by using the detrending algorithm, while 11 feature wavelengths extracted by variable combination population analysis (VCPA) method effectively contained characteristic group information of linoleic acid. The Bi-directional LSTM (Bi-LSTM) model combined with the feature extraction data set of VCPA method predicted 0.860 Rp^2^ value of linoleic acid content in red meat. The CNN-Bi-LSTM model achieved an Rp^2^ of 0.889, and the optimized Bayes-CNN-Bi-LSTM model was constructed to achieve the best prediction with an Rp^2^ of 0.909. This study provided a reference for the rapid synchronous detection of mixed sample indicators, and a theoretical basis for the development of hyperspectral on-line detection equipment.

## 1. Introduction

Red meat, a key source of high-quality protein, myoglobin, fatty acids, and iron, plays a crucial role in the production of meat products [1]. Among the fatty acids, unsaturated ones are categorized into two primary types: monounsaturated and polyunsaturated. These unsaturated fatty acids in red meat contribute to lowering cholesterol levels and enhancing cardiovascular health. However, its higher saturated fatty acid content raises concerns regarding human health. Reducing the saturated fatty acid content of red meat and analyzing the species types of unsaturated fatty acid has become an important development trend in premium meat processing [2]. The polyunsaturated fatty acid (PUFA) content in red meat accounts for about 10–20% of its total fatty acids, with linoleic acid being the most abundant [3]. Linoleic acid, an essential PUFA, serves as a vital precursor for synthesizing key substances such as docosahexaenoic acid (DHA) and eicosapentaenoic acid (EPA) in the human body. It plays a pivotal role in regulating inflammation and immune functions [4], which are essential for human growth and development. It is of great significance to analyze and evaluate the linoleic acid content in meat products by using rapid non-destructive testing technology for fresh meat processing.

Current methods for quantitative linoleic acid detection are mostly rely on gas chromatography [5]. Although, these methods offer high detection accuracy, but their operation is time-consuming and labor-intensive to meet the demand of modern meat processing industry due to speed and convenience [6]. In recent years, few rapid detection techniques have been continuously applied for the analysis of meat quality [7], including near-infrared spectroscopy, Fourier infrared, and nuclear magnetic resonance [8,9]. However, these methods primarily focus on quantifying the fatty acid content without providing spatial information on their distribution within the samples [10,11]. This gap can be effectively bridged by HSI technology, which combines computer vision with spectroscopic analysis. HSI can collect the spectral information from each pixel position of the sample in hundreds of continuous spectral bands, enabling both quantitative analysis and visualization of the spatial distribution of fatty acids. It establishes the quantitative analysis relationship between the components to be measured and the spectral absorption response by using characteristic wavelength groups and octave frequencies [12]. HSI plays a significant role in the field of modern agricultural products detection [13,14,15]. Therefore, this study employs near-infrared HSI technology to conduct a rapid detection of linoleic acid content in red meat.

Linoleic acid is characterized by its identical molecular groups and carbon chain structure in the red meat, and exhibits analogous composition to the components and muscle fiber structure of red meat. The development of a spectroscopic method for the detection of red meat coupled with the construction of a chemometrics analytical model holds significant prospective value for the advancement of fresh meat industry [16,17]. This study was focused on detecting linoleic acid content in mixed red meat from various cultivars, locations, and species and on constructing highly robust predictive models. Deep neural networks were extensively utilized in the model building analysis due to their capacity for automatic feature extraction, high robustness, and handling of data redundancy [18].

In this study, the largest consumption of cattle, sheep and pork by Chinese residents was taken as the research object, and the visible near-infrared hyperspectral (400–1000 nm) fusion deep learning optimization algorithm was used for rapid detection of linoleic acid content in red meat [19]. The LSTM model and a Bi-LSTM model were implemented to extract the deep linoleic acid from both forward and reverse regression prediction data [20]. Then, a two-layer convolutional neural network (CNN) decision layer optimization method was used to build a CNN-Bi-LSTM model, aligning with method described by Li, et al. [21,22]. Bayesian algorithm was used to optimize the model optimal hyper-parameters and the Bayes-CNN-Bi-LSTM was used to improve the linoleic acid content prediction model [23]. This research provided a theoretical basis for the development of rapid meat quality testing and online testing equipment.

**Figure 1:**
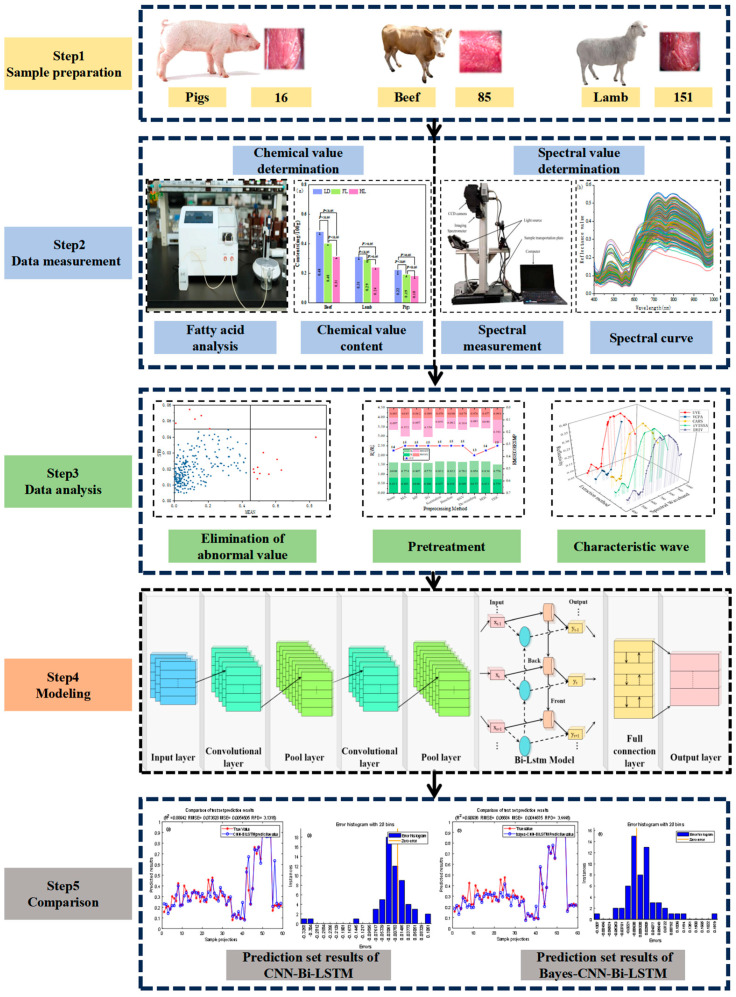
Key steps of the experiment.

In this study, samples of beef, lamb and pork were purchased from ethical abattoirs in the Northwest Territories, and the specific research process was started by collecting the hyperspectral band ranging from 400–1000 nm in red meat samples. This process allowed for the fusion of information to determine the linoleic acid content in the red meat using gas chromatography. Segmented thresholding method was employed to construct mask images, selecting regions of interest within the red meat samples. This facilitated the extraction of corresponding regional spectral information. The most effective preprocessing method was applied depending on Partial Least Square Regression (PLSR) model and wavelength optimization methods to extract the five feature wavelengths. After then, the CNN decision layer fusion optimization method was used to establish a CNN-Bi-LSTM model to predict the linoleic acid content in red meat samples. Then, Bayesian optimization algorithm was used to find the optimal hyperparameters of the model and established the Bayes-CNN-Bi-LSTM model, which improved the prediction accuracy of the linoleic acid content in red meat. The key steps of the experiment are shown in Figure 1.

## 2. Materials and Methods

### 2.1. Sample Preparation

The experiment was conducted with a variety of livestock, including 50 sheep (35 Ningxia Tan lamb and 15 Gansu small-tailed cold sheep), 20 yellow cattle (comprising 12 Angus, 8 Simmental breeds), and 8 Duroc pigs. A total of 252 samples were collected, including 151 mutton samples (divided into 57 longissimus dorsi muscle (LD), 42 foreleg (FL), and 52 hindleg (HL)), 85 yellow beef samples (23 LD, 32 FL and 30 HL), and 16 pork samples (8 LD, 4 FL and 4 HL). After purchasing all the required samples from ethically qualified abattoirs, the samples underwent acid excretion 4 °C for 48 h. After acid excretion, the LD, FL, and HL cuts of red meat were processed to remove excess fat and oleic acid from the surface. The carcasses were vacuum sealed and transported to the laboratory for storage at 4 °C prior to the experiment. To maintain the consistency of the samples, the red meat cuts were standardized into small pieces measuring 35 mm × 25 mm × 10 mm. Prior to spectral scanning, samples reached center temperature at 20 °C, the excess water was absorbed using filter paper.

### 2.2. Determination of Chemical Values

Fatty acid content was determined by the Floch method, and experiments were carried out according to procedure conducted by Fleming, et al. [24]. The fatty acid profile of the procured samples was analyzed using a GC equipped with a flame ionization detector (FID) [24,25]. A homogeneous sample ranging from 0.1 g~10 g was weighed to a precision of 0.1 mg and placed into a 250 mL flat-bottomed flask. Subsequently, 2.0 mL of triglycerol undecanoate internal standard solution was added to the flask. About 100 mg of pyrogallic gallic acid and a few grains of zeolite were added to assist the reaction. Then, 2 mL of 95% ethanol, 4 mL of water, and 10 mL of hydrochloric acid solution was added into the above flat-bottomed flask, and eventually mixed well [26]. The methyl esters (C11:0, procured from Sigma Aldrich Pty Ltd., Castle Hill, NSW 2154, Australia) of fatty acids were derived using a 10 N KOH solution in methanol. The flask was hydrolyzed in a water bath at 70 °C~80 °C for 40 min, and the flask was shaken every 10 min to mix the particles adhering to the flask wall into the solution. After hydrolysis, the flask was removed and cooled at room temperature. Then, 8 mL of 2% sodium hydroxide methanol solution was added to the fat extract, followed by the attachment of reflux condenser. The mixture was refluxed on a water bath at 80 °C ± 1 °C until the oil droplets disappeared. Next, 7 mL of 15% boron trifluoride methanol solution was added from the upper end of the reflux condenser, and the mixture was continued to reflux for 2 min at 80 °C ± 1 °C. The reflux condenser was rinsed with a small amount of water. The heating was stopped, and the flask was removed from the water bath, and quickly cooled to room temperature. Accurately, 10 mL–30 mL n-heptane was added and shaken for 2 min. Then, saturated aqueous sodium chloride solution was added, and the mixture was set to stratify. The upper layer of n-heptane extraction solution about (5 mL to 25 mL) was pipetted into a test tube. About 3–5 g of anhydrous sodium sulfate was added and shaken for 1 min. The mixture was allowed to stand for 5 min, and the upper layer of the solution was pipetted into the injection bottle to be measured. To mitigate experimental errors and obtain a mean value, this process was repeated three times.

### 2.3. Acquisition and Calibration of Hyperspectral Images

Hyperspectral images of the red meat samples were acquired using a HSI system. The HSI system consisted of a spectrograph (Spectral Imaging Ltd., Oulu, Finland), a charge-coupled device (CCD) camera (model XC-130100HZ, Ophir Optoelectronic Solutions Ltd., Jerusalem), an optical system consisting of a stepper motor (PSA200-11-X, Zolix Instrument Co. Ltd, Beijing, China), four tungsten-halogen lamps (50 W) as light sources, and a computerized system using Spectral Cube software. A range of 400–1000 nm was used for sample hyperspectral image acquisition.

The Hyperspectral Imaging (HSI) system discussed in the paper has an acquisition range of 400–1000 nm, with the spectral resolution of the acquired hyperspectral images being 2.8 nm across 125 bands. Each sample was placed on a black background and scanned line by line [27]. After several trials, the moving speed of the conveyor platform was set to 20 mm/s to avoid image distortion. The distance between the light source and the samples was set to 40 mm, and the exposure time was set to 3 ms. Correction of the raw images was required to eliminate the dark current effect of the camera and to eliminate the effect of uneven illumination. A white reference image was obtained from a white reference panel (100% reflectivity) and a dark reference image was obtained by covering the camera (0% reflectivity). The correction was performed using the following formula:

(1) $$I(\%)=\frac{R-R_{d}}{R_{w}-R_{d}}\times 100\%$$

where I represents the obtained calibrated image, R represents the acquired original image. $R_{d}$ represents the completely black image, $R_{w}$ represents the white reference image.

### 2.4. Extraction of Region of Interest and Division of the Data Set

In this study, a small rectangular block area in the image, which minimizes the inclusion of fat, was selected as the region of interest (ROI) using the rectangular region method. The extraction of the Region of Interest is a fundamental technique in image processing and computer vision. It is employed to designate specific portions of an image for more precise and efficient analysis and processing. Segmentation calibration set prediction, a form of training dataset segmentation during machine learning training, is intended to evaluate the performance of a model on the data that it has not previously encountered. The spectral data were divided into correction and prediction sets in the ratio of 3:1 by KS (Kennard-Stone), RS (Random Sampling) and SPXY (Sample Set Partitioning based on joint X-Y distances) after removing the abnormal samples by Monte Carlo method. A total of 183 correction sets and 61 prediction sets were obtained [28]. The KS method follows a step-by-step process, ensuring that the results of sample set partitioning are uniformly distributed in the spectral data space. The SPXY method considers the influence of spectral variables and physical and chemical reference values of the samples during the sample set partitioning process. This ensures that the samples in the correction set have a more effective multidimensional spatial distribution, thereby enhancing the predictive ability and robustness of the correction model. The RS method involves randomly selecting a specified number of samples from the sample set to form the correction set. The RS method randomly selects a specified number of samples from the overall sample set to form the correction set, with the remaining samples forming the test set. Predictive models for different segmentation methods were developed to select the optimal method. A reasonable sample set is the basis for establishing a quantitative analysis model, and the division of the sample set determines the superiority of the constructed model to a certain extent. The chemical and spectral values of the samples selected in the correction set should be representative when dividing the samples but should not lead to data redundancy and model overfitting [29].

### 2.5. Spectral Preprocessing and Selection of Characteristic Wavelengths

Nine preprocessing algorithms were used in this study to optimize the spectral data and eliminate disturbances such as baseline drift and noise. Convolutional smoothing constructs a digital filter function using the least squares fitting coefficients to achieve convolutional smoothing of the original data. The primary purpose of the centering method is to alter its spatial coordinates and origin. Normalization processing primarily corrects the scaling problem of the sample spectral variables caused by the influence of the optical signal path, scattering, and changes in the light source. The baseline calibration method eliminates background noise, thereby making the signal easier to interpret. The Standard Normal Variable Transform algorithm eliminates spectral errors due to light scattering and light range changes. The De-trending method removes baseline drift from the spectrum. The multiple scattering correction algorithm initially takes the average value of the sample spectra of the correction set as the baseline, and then performs a one-dimensional linear regression operation on all the spectra and the baseline spectra to realize the original spectral preprocessing analysis sequentially. The orthogonal signal correction method can effectively remove the useless information in the original spectra that has less correlation with the physical and chemical indices to be measured. The optimal preprocessing algorithm was selected by comparing the model performance of different methods following the procedure described in the reference with slight modifications [30].

The full-band spectra contains a large amount of data and redundant information, which leads to a decrease in computational speed during the modeling process [31]. To eliminate irrelevant information, realize data dimensionality reduction, and improve computational efficiency, the wavelength that represents the most spectral information of the measured samples was selected [32]. In this study, the following characteristic wavelength selection algorithms were used: Iterative Retention of Information Variables is an iterative-based information variable extraction method that reduces the complexity of the model and improves spectral detection accuracy. Competitive Adaptive Re-weighting Algorithm is an algorithm that uses Monte Carlo sampling and PLS regression coefficients. Variable combination cluster analysis iteratively shrinks the variable space by applying an exponential decay function to obtain the variable combination with the lowest RMSECV. Interval Variable Iterative Space Shrinkage is a novel feature wavelength extraction algorithm proposed based on the variable iterative space shrinkage method. It optimizes and analyzes the position, width, and combination of spectral intervals using methods such as iteration and intelligent preference. The uninformative variable elimination method can remove the wavelength modeling that contains more noise and less relevant information in the spectra, thus effectively avoiding model overfitting.

### 2.6. Model Construction

PLSR model, LSTM model and Bi-LSTM model for linoleic acid content in red meat were developed, aligning with models established by Naganathan, et al. [33], and then Bi-LSTM model was compared and contrasted. The CNN-Bi-LSTM model was established using the concept of optimized fusion of decision layers of convolutional neural networks. This was followed by the Bayesian algorithm optimized Bayes-CNN-Bi-LSTM combined model to predict the linoleic acid content in red meat.

#### 2.6.1. LSTM Networks and Bi-LSTM Networks

**Figure 2:**
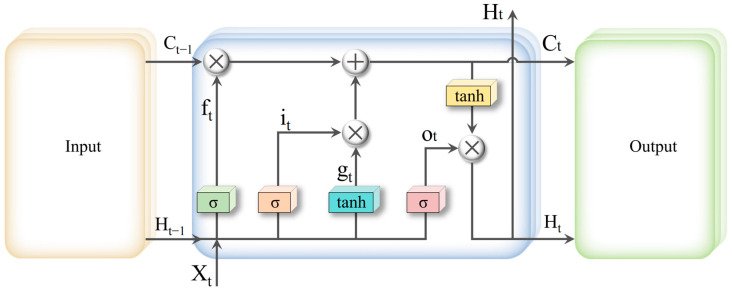
Structure of LSTM cell.

**Figure 3:**
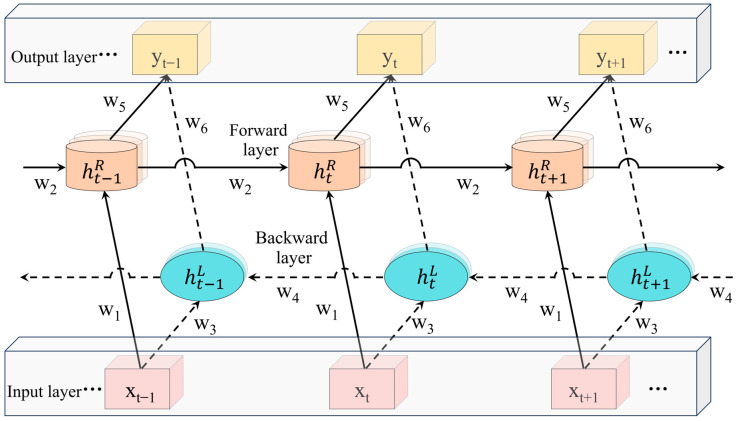
Structure of Bi-LSTM cell.

PLSR is a generalization of multiple linear regression and is most commonly used among the partial least squares algorithms [34]. LSTM is an improvement of Recurrent Neural Networks (RNN) [35]. LSTM effectively screens and updates information, addressing the issue of gradient disappearance that can occur when processing sequence or multivariate regression data in RNN by introducing gate functions (forgetting gate, input gate, output gate) and memory units on the hidden layer of RNN. The internal structure of a single LSTM cell of the LSTM layer is shown in Figure 2. Bi-LSTM is an improvement of LSTM network, comprising a forward LSTM layer and a backward LSTM layer [36] (Figure 3). Unlike LSTM, which can only learn features in a single direction, Bi-LSTM enables bi-directional feature learning on data. This allows for better acquisition of the correlation between the features of multivariate regression data; hence Bi-LSTM is introduced as the feature learning unit of the CNN-Bi-LSTM model.

Figure 2 illustrates the structure of a typical LSTM network. It is capable of analyzing data with strong linearity and noise, handling a large number of X-variables, and modeling multiple response variables simultaneously. For the neuron structure at the current moment ‘t’, there were three input values as explained [37]: the neuron cell state ‘$C_{t-1}$’ in the previous moment, the neuron output ‘$H_{t-1}$’ in the previous moment and the input ‘$X_{t}$’ to the network at the current moment. Moreover, there are two output values: the neuron cell state ‘$C_{t}$’ at the current moment and the current network output value ‘$H_{t}$’.

In this study, each LSTM neuron structure also included three gate structures: the oblivion gate ‘$f_{t}$’ controlled the number of neuron states from the previous moment into the current unit, the input gate ‘$i_{t}$’ controlled the number of network inputs entering the cell at the current moment, and the output gate ‘$o_{t}$’ controlled the number of cell state entering the current cell output. The candidate state of the unit at the current moment is denoted as ‘$g_{t}$’.

The outputs of the forward and backward layers at the moment ‘t’ are denoted as ‘$h_{t}^{R}$’ and ‘$h_{t}^{L}$’, respectively. The weight matrices between the input layer and the forward layer and the backward layer are represented as ‘$W_{1}$’ and ‘$W_{3}$’, respectively. The weight matrix between the forward-propagating unit at the previous moment and the current unit is denoted as ‘$W_{2}$’. The weight matrix between the backward propagation unit and the current unit at the next moment is represented as ‘$W_{4}$’. the weight matrices between the forward layer, the backward layer and the output layer are denoted as ‘$W_{5}$’ and ‘$W_{6}$’, respectively.

#### 2.6.2. The Decision Layer Fusion Network Modeling Framework of the CNN and CNN-Bi-LSTM

Conventional neural network are feedforwarding neural network that incorporate convolutional operations and have a deep structure [38]. The convolutional layer used a convolutional kernel to convolutional the input data on the local region, generating the corresponding features. The pooling layer down sampled these features to achieve data dimensionality reduction. The fully connected layer transformed the extracted local features into feature vectors, which passed to the Bi-LSTM neural network for prediction: The prediction is computed using the equation as explained [39,40,41]:

(2) $$c_{i}=f(w\times x_{i:i+g-1}+b)$$

In this equation, ‘w’ represents the convolution kernel, ‘g’ is the size of the convolution kernel, ‘$x_{i:i+g-1}$’ is the feature vector from ‘i’ to ‘i+g−1′. Where ‘b’ is the bias term. This process resulted in the feature matrix ‘G’ is obtained, which is represented as G=[c1,c2,……,c6].

#### 2.6.3. Bayesian Algorithm Optimization

A convolutional neural network “CNN” model cannot be optimally generalized to all datasets. Therefore, it’s important to select an appropriate set of hyperparameters for the CNN before applying it to a new dataset. In this study, hyperparameters included the number of layers, the activation function for each layer of hidden units, the kernel size of the layer, the configuration of layers within the network, etc. Selecting a new model for a new dataset could be a time-consuming and tedious task. However, the optimization of hyperparameters was viewed as the optimization of an unknown black-box function that reflected the generalization performance. Bayesian optimization provided an effective approach and has been shown to outperform other state-of-the-art global optimization algorithms on many challenging optimization benchmark functions. The whole process was carried out as previously explained by Liu, et al. [42].

### 2.7. Modeling Evaluation

The quantitative analysis model, encompassing both linear and artificial neural networks, was constructed using the CNN-Bi-LSTM model, which integrates PLSR, CNN and Bi-LSTM. The quantitative analysis model performance was evaluated using several metrics, including the calibration set coefficient of determination (Rc^2^), prediction set coefficient of determination (Rp^2^), mean absolute error (MAE), root mean square error of prediction set (RMSEP), mean squared error (MSE), root mean squared error (RMSE), mean absolute percentage error (MAPE), standard deviation of the prediction (SEP), and relative analysis error (RPD) [43]. Generally, a robust model is characterized by higher R^2^ and RPD values and lower RMSE values. This indicates that the model is accurately predicting the outcomes and the discrepancy between the predicted and actual values is minimal [44].

### 2.8. Data Analysis

ENVI 4.8 software (ITT Visual Information Solutions, USA) was employed to extract original spectral data. The Unscrambler-X 10.4 software was used to construct the PLSR model. Python 1.10 software was utilized to build the LSTM and Bi-LSTM model. Matlab R2020a software (Myswark Software, Beijing, China) was used to divide the sample set and establish the Bayes-CNN-Bi-LSTM model. And the data were plotted for graphing using Origin 2021.

## 3. Results and Discussion

### 3.1. Chemical Values and Spectral Curves

**Figure 4:**
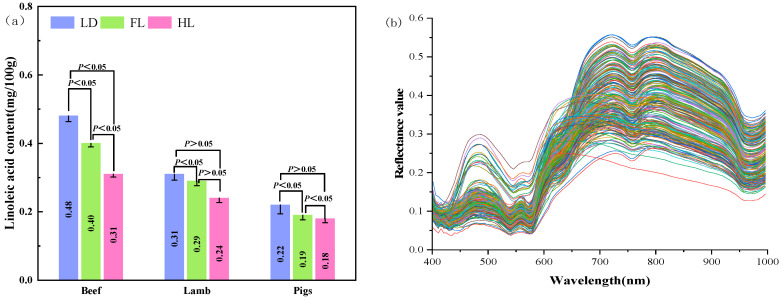
Chemical values and spectral curves of linoleic acid content of red meat. They should be listed as: (**a**) Linoleic acid content in Ningxia Tan lamb, yellow beef and Duroc pork. (**b**) The spectral curves of the Ningxia Tan lamb, yellow beef, and Duroc pork.

A gradual decrease was observed in the distribution of linoleic acid content from LL to FL and HL in yellow beef, Ningxia Tan sheep, and Duroc pork, as determined by the GC method (Figure 4a). This trend may reflect greater exercise of the FL and HL compared to the LD, resulting in lower fat content and linolenic acid levels, as the LD had less movement and therefore accumulated fat content. The significant exercise-induced increase in aerobic metabolism of the FL and HL is consistent with their lower linoleic acid content compared to LD, with no significant difference (*p* < 0.015) between the HL and the LD. Meanwhile, it has been shown that muscle tissues with lower fat content have higher levels of PUFA [45].

The raw spectral images of the LD, FL, and HL of the red meat samples (Figure 4b) demonstrated similar spectral curves for beef and lamb, distinct from pork spectra. The colored lines in the figure refer to the curve of spectral wavelength versus reflectance. Beef and lamb spectra exhibited peaks at 479, 723, and 799 nm and troughs at 429, 540, and 759 nm, while the pork spectra showed two peaks at 479 and 618 nm and troughs at 429 and 548 nm. The troughs near 429 and 540 nm may be related to oxyhemoglobin absorption [46], while peaks near 479, 723, and 799 nm may be derived from -NH3- groups in the protein fractions as well as the combined absorption band of C-H and C-O in the fat [47]. Lastly, the spectral peaks were characterized by the 900–1000 nm wavelength water absorption bands [48,49]. These spectral differences can be used for subsequent prediction of linoleic acid content in different types of red meat.

Reduction in linoleic acid content from the LD to FL to HL cuts was observed in beef cattle, and suggested this was linked to differences in intramuscular fat content and metabolic activity [50]. Similar spectral absorption peaks and troughs in beef was characterized and associated with myoglobin, fat, and moisture content. They developed prediction models for beef composition using these spectral features [51]. Spectral techniques including near-infrared spectroscopy (NIRS), HSI, and RS was employed for the assessment of meat [52].

### 3.2. Outlier Detection and Division Prediction

**Figure 5:**
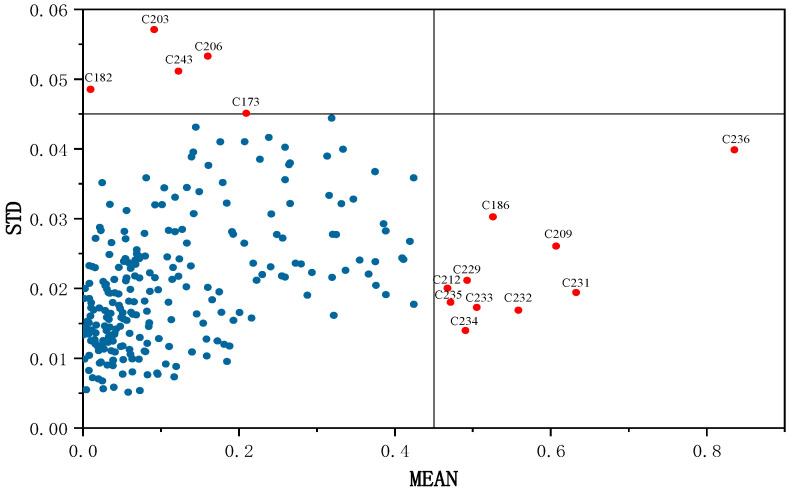
Rejection of the abnormal sample-based MC method.

Monte Carlo (MC) method, commonly used for outlier elimination, aids in distinguishing between samples and enhancing model accuracy. This method falls under the category of abnormal sample identification methods based on Monte Carlo Cross Validation (MCCV). MCCV is used to randomly partition the correction set and prediction set. It primarily employs a random sampling method to calculate the corresponding mean and standard deviation, which are used to plot the distribution. The impact of outlier removal on model prediction has been studied extensively. This approach aids in distinguishing differences among the samples and enhancing the model’s accuracy. The rule of this method involves setting a threshold; any simulation result exceeding this threshold is deemed an outlier. Previous research showed that by removing influential points or outliers, the model’s adequacy can be increased [53]. Points significantly distant from the main sample are typically considered outliers and are eliminated [54]. Initially, all samples are considered as part of correction set, and loop modeling is performed multiple times to obtain various prediction errors for each sample. The modeling effect of each outlier was obtained by removing samples. From a total of 252 samples, the MC method identified 15 outliers (183, 203, 243, 206, 173, 233, 186, 232, 209, 231, 236, 212, 235, 229, 234). The test yielded a map of the distribution of samples detected with abnormal linoleic acid content in red meat (Figure 5). The blue dots in the graph indicate the distribution of linoleic acid content in each red meat sample. However, the predictive performance of the PLSR model declined when samples 233, 186, 232, 212, 235, 229, and 234 were removed, leading to their retention. In this study, the minimum RMSE was used to determine the number of latent variables during the 10-fold cross-validation process [55]. As a result, the Rcv^2^ value increased from 0.691 to 0.786, and the RMSEP value decreased from 0.118 to 0.098. The modeling results of the MC method for detecting abnormal red meat samples are shown in Table 1.

After outlier removal, 183 samples were selected as the calibration set and 61 samples as the prediction set using the KS, RS and SPXY algorithms. The calibration set encompasses the range of the linoleic acid content prediction set, and the minimal difference between the mean and standard deviation of the two data sets suggests similar distributions of the divided sample sets. This is very conducive for constructing a prediction model with high accuracy and robustness. Meanwhile, when comparing the regression prediction models using PLSR method under KS, RS and SPXY algorithms, the modeling prediction effect of RS division correction prediction was the best, with Rp^2^ reaching 0.808 (Table 2). The RS method is the optimal strategy for partitioning the sample set when predicting linoleic acid content in red meat. The RS method operates by randomly selecting a specified number of samples from the total sample set to form the calibration set, with the remaining samples constituting the test set. Given its random selection process, the RS method is particularly suitable for predicting the linoleic acid content in red meat. In this experiment, where the linoleic acid content in mixed red meat from multiple species, breeds, and cuts was predicted, the RS method proved to be the most appropriate choice for randomly selecting the calibration set. The use of KS, RS, and SPXY algorithms in model prediction has been documented in several studies [56,57]. These algorithms are used for partitioning training and validation sets in multivariate modeling. The automatic scaling of the response variables in the PLSR model is employed, which is a feature of gradient-based methods. More specifically, we used a back-propagation algorithm to compute the gradient of each response variable. This gradient was then used to scale the response variable, effectively reducing the risk of overfitting. This approach not only improves the model’s generalization ability but also ensures its accuracy.

### 3.3. Spectral Data Preprocessing Methods

**Figure 6:**
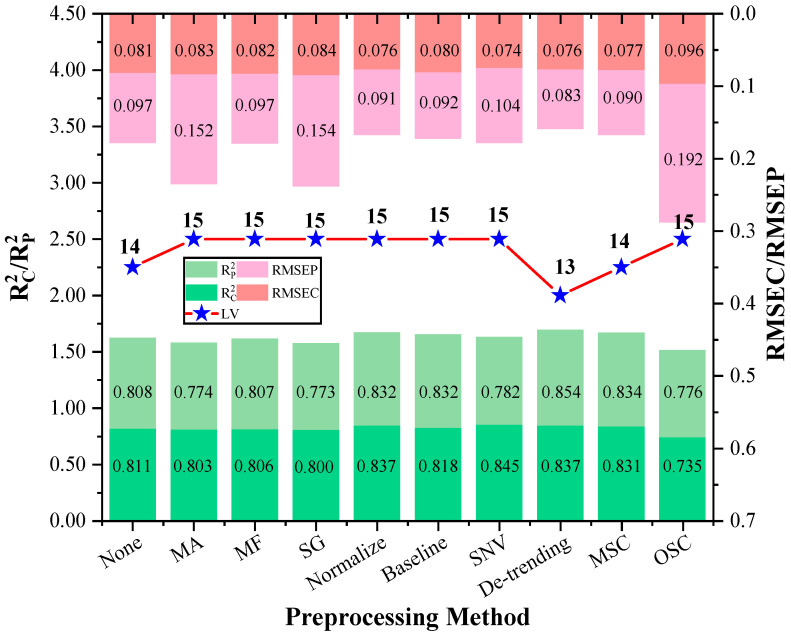
Comparison of statistical results of preprocessed sample sets.

The resolution of overlapping data was enhanced and the system noise was reduced by spectral scattering and instrumental drift. This paper evaluated and compared nine preprocessing methods based on the PLSR model for full-wavelength (Figure 6). The comparison revealed a significant improvement in the modeling effect of the PLSR model post preprocessing. Among the methods evaluated, the De-trending algorithm as a nonlinear signal processing technique demonstrated superior filtering effect on spectral images. De-trending preprocessing method proved to be more stable, with an Rp^2^ value of 0.834, and a root mean square error prediction value of 0.090. Compared to the original model, Rp^2^ value improved by 0.173, indicating the effectiveness of this method. The Detrending Moving Average (DMA) algorithm has been widely used in its several variants for characterizing long-range correlations of random signals and sets (one-dimensional sequences or high-dimensional arrays) over regression. In a previous study, the scaling performances of the centered DMA mainly based on analytical arguments investigated by means of a continuous regression approximation and a frequency response approach [58]. A study represented a comprehensive view on machine learning algorithms that was applied to enhance the intelligence and the capabilities of an application [59]. Multivariate regression data was used to create a new dataset where each observation had the difference between itself and the previous observation [60].

### 3.4. Modeling of Featured Wavelength Extraction

**Figure 7:**
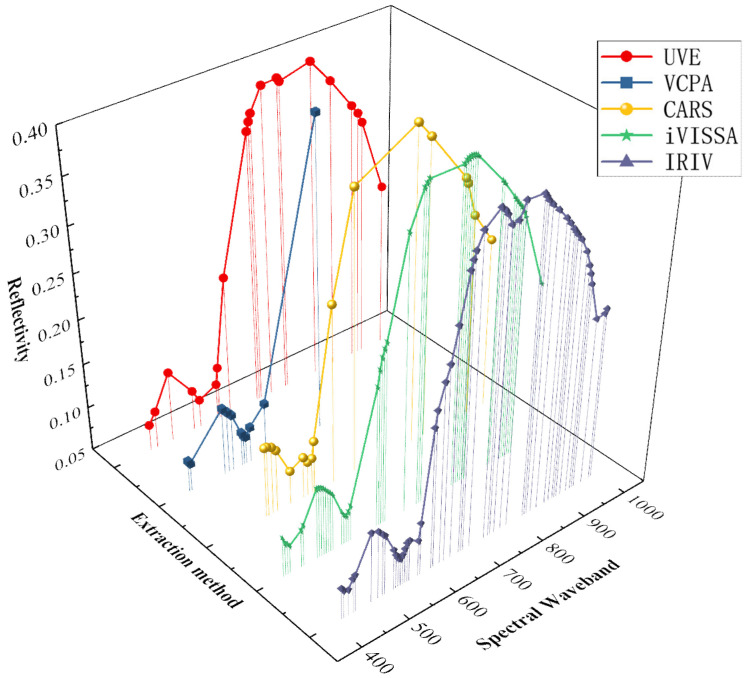
Comparison of five feature wavelength extraction methods.

The feature variable screening method based on the weighting algorithm was selected to evaluate the full data and local area data of linoleic acid to improve the prediction performance of the model. The aim was to verify the influence of different weighting methods on the model effect. In this study, UVE, VCPA, CARS, iVISSA, and IRIV algorithms were used to extract the characteristic wavelengths. Plot and compare the distribution curves of the five characteristic wavelength extractions (Figure 7).

Under the De-trending preprocessing method, PLSR, LSTM, and Bi-LSTM models were established for linoleic acid to compare different feature band extraction methods. The linear regression modeling approach has low performance in predicting linoleic acid content in red meat, while the Bi-LSTM model achieves both high performance in predicting linoleic acid content in red meat with fewer eigen bands. The UVE method extracted 20 feature bands, achieving a better modeling effect with Rc^2^ and Rp^2^ values of 0.686 and 0.750, respectively. The VCPA and iVISSA methods extracted 11 and 45 feature wavelengths, respectively, resulting in comparable model effects. The IRIV method offered higher model accuracy, extracting 52 feature wavelengths with Rc^2^ = 0.794, RMSEC = 0.085, SEC = 0.085; Rp^2^ = 0.840, RMSEP = 0.092, SEP = 0.093. However, this method extracted a large number of feature wavelengths. In the LSTM model and Bi-LSTM models, the best results were achieved with the Bi-LSTM model under the VCPA feature extraction method. The results of the feature wavelength extraction modeling are presented in Supplementary Materials with an Rp^2^ enhancement of 0.860, an MSE of 0.013, an RMSE of 0.1154, and an MAE of 0.073. The results of the feature wavelength extraction modeling are presented in Table 3 and Table S1.

### 3.5. CNN-Bi-LSTM Network Model and Bayes-CNN-Bi-LSTM Model Framework

Different species and varieties of red meat samples contain large background variability, and the distribution of linoleic acid content has strong stochastic and nonlinear characteristics. In this research work, the deep learning optimization algorithm was employed for the analysis. CNN-LSTM model demonstrated strong feature extraction ability and excellent multivariate regression processing ability [61]. This experiment combined a variety of multivariate regression feature sensitive models and improved the CNN-Bi-LSTM model to construct the CNN-Bi-LSTM model for analyzing the distribution of linoleic acid content in red meat. The original spectral data of linoleic acid was mapped to the hidden feature data for feature extraction. This process reduced the loss of information from the extracted features in the fully connected layer, thereby optimizing the prediction effect. After optimization, the CNN-Bi-LSTM model yielded an Rp^2^ of 0.889, the RMSE of 0.074, the MSE of 0.043, the MAE of 0.043, the MAPE of 0.176, and the RPD of 3.131. This result indicated an improvement of 0.08 compared to the LSTM model, and 0.029 compared to the Bi-LSTM model. The CNN-Bi-LSTM model demonstrated the better performance in predicting the linoleic acid content of red meat. The parameters of the modeling process contain LSTM model parameters, CNN model parameters, and Bayes parameters have been enumerated (Tables S2 and S3).

**Figure 8:**
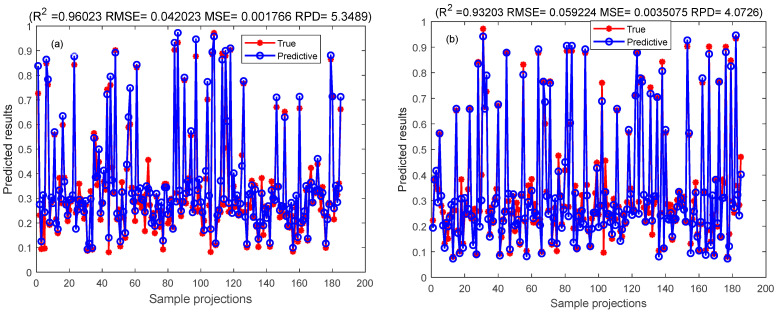
Comparison of calibration set results of CNN-Bi-LSTM model and Bayes-CNN-Bi-LSTM model. They should be listed as: (**a**) Comparison map of the prediction results of the CNN-Bi-LSTM model calibration set; (**b**) Comparison of the prediction results of the Bayes-CNN-Bi-LSTM model calibration set.

**Figure 9:**
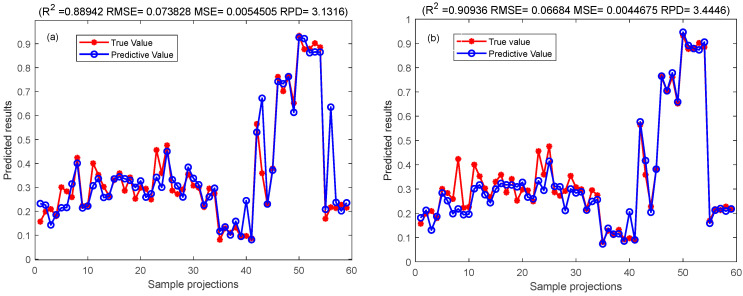
Comparison of prediction set results of CNN-Bi-LSTM model and Bayes-CNN-Bi-LSTM model. They should be listed as: (**a**) Comparison map of the prediction results of the CNN-Bi-LSTM model test set; (**b**) Comparison of the prediction results of the Bayes-CNN-Bi-LSTM model test set.

**Figure 10:**
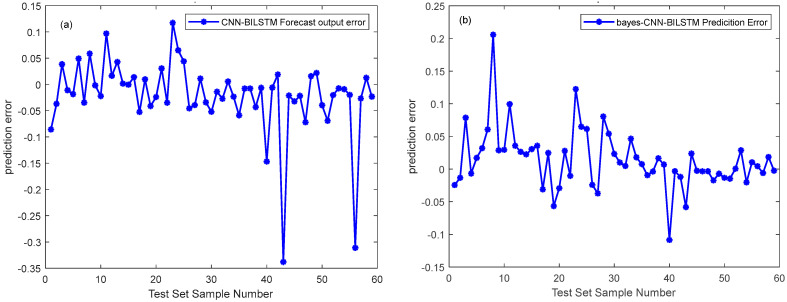
Comparison of prediction error between CNN-Bi-LSTM model and Bayes-CNN-Bi-LSTM model predictions set. They should be listed as: (**a**) Prediction error map of the CNN-Bi-LSTM model test set; (**b**) Prediction error map of the Bayes-CNN-Bi-LSTM model test set.

**Figure 11:**
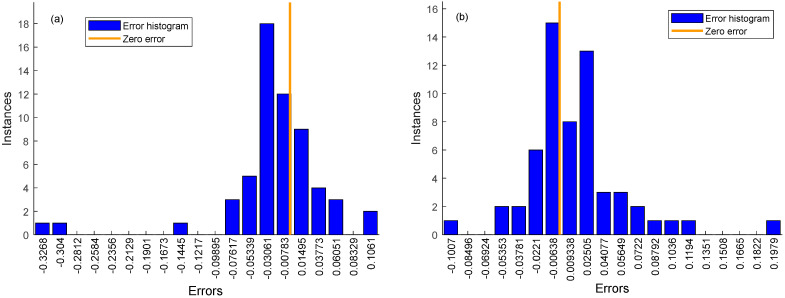
Error histogram of CNN-Bi-LSTM model and Bayes-CNN-Bi-LSTM model with 20 bins.they should be listed as: (**a**) Error histogram of CNN-Bi-LSTM model with 20 bins; (**b**) Error histogram of Bayes-CNN-Bi-LSTM model with 20 bins.

Generalization of all datasets was optimized to solve the phenomenon of overfitting in the prediction process of CNN-Bi-LSTM model. The Bayesian algorithm was employed to optimize the unknown objective function, and the Gaussian process was used to accelerate the iterative search function for the optimal solution. This approach facilitated the quick determination of the optimal solution, and the Bayes-CNN-Bi-LSTM model quickly was created to further improve the prediction effect of linoleic acid content in red meat [62]. The results of Bi-LSTM model and Bayes-CNN-Bi-LSTM model results are compared as shown in Table 4 with corresponding figures presented in Figure 8, Figure 9, Figure 10 and Figure 11, respectively.

The study established two models, the CNN-Bi-LSTM model and Bayes-CNN-Bi-LSTM, for predicting the linoleic acid content in red meat. These models were developed based on the distribution of linoleic acid content and a feature wavelength extraction method. Table 4 presents the prediction results of the CNN-Bi-LSTM model and Bayes-CNN-Bi-LSTM model. The Bayes-CNN-Bi-LSTM model outperformed the CNN-Bi-LSTM model, achieving an Rp^2^ value exceeding 0.909 and an RMSE less than 0.074. This indicated that the Bayes-CNN-Bi-LSTM model predicted the linoleic acid content in red meat more effectively. Moreover, the model quickly identified the optimal hyper-parameters, demonstrating high robustness and accuracy. Therefore, the Bayes-CNN-Bi-LSTM model proposed in this study is suitable for comprehensive evaluation and prediction of linoleic acid content in red meat.

## 4. Conclusions

HSI was utilized in conjunction with deep learning optimization algorithm to predict and analyze the linoleic acid content in 252 red meat samples. These samples were sourced from multi-species, varieties, and parts. The results indicated that the Bi-LSTM model combined with the dataset extracted using the features of the VCPA method, could effectively predict the linoleic acid content in red meat, achieving a predicted Rp^2^ of 0.860. The bi-layer CNN decision-layer optimization approach to build the CNN-Bi-LSTM model further improved the prediction effect, with the Rp^2^ reaching 0.889. Finally, the Bayesian algorithm was employed to optimize the hyperparameters of the model, reducing overfitting and enhancing the robustness of the model. In this study, The De-trending-VCPA-Bayes-CNN-Bi-LSTM model was created to predict the linoleic acid content of red meat to achieve the best effect, with an Rp^2^ of 0.909. This study can serve as a reference for rapid and synchronous detection in multi-species, multi-species, multi-site fusion samples. The related modeling method further expands the application field and accuracy of HSI technology in meat quality analysis, providing a theoretical basis for the development of hyperspectral on-line detection equipment.

## Figures and Tables

**Table 1:** Predictions results of abnormal red meat samples based on MC method.

Samples Removal	Number of Samples	LVs	Calibration Set		Verification Set	
			Rc^2^	RMSEC	Rcv^2^	RMSECV
Before elimination	252	16	0.724	0.111	0.691	0.118
After elimination	244	12	0.805	0.087	0.786	0.098

**Table 2:** PLSR modeling results of linoleic acid content of red meat based on three different division.

Datasets	Sample	Partition Method	LVs	R^2^	RMSE	SE
Calibration set	183	KS	15	0.794	0.097	0.097
		SPXY	10	0.789	0.098	0.098
		RS	14	0.811	0.081	0.082
Prediction set	61	KS	15	0.700	0.072	0.071
		SPXY	10	0.739	0.060	0.060
		RS	14	0.808	0.097	0.098

**Table 3:** Statistical results of characteristic wavelength extraction.

Method	Partition Method	Numbers	Calibration Set				Prediction Set			
			Rc^2^	MSE	RMSE	MAE	Rp^2^	MSE	RMSE	MAE
LSTM	UVE	20	0.720	0.057	0.238	0.221	0.757	0.009	0.087	0.071
	VCPA	11	0.821	0.072	0.269	0.256	0.809	0.020	0.109	0.075
	CARS	17	0.750	0.056	0.237	0.220	0.776	0.007	0.084	0.058
	iVISSA	45	0.756	0.063	0.250	0.236	0.740	0.011	0.107	0.072
	IRIV	52	0.724	0.077	0.277	0.261	0.745	0.008	0.093	0.065
Bi-LSTM	UVE	20	0.774	0.075	0.230	0.210	0.787	0.007	0.086	0.062
	VCPA	11	0.846	0.064	0.253	0.233	0.860	0.013	0.115	0.073
	CARS	17	0.790	0.076	0.276	0.260	0.810	0.007	0.085	0.064
	iVISSA	45	0.742	0.074	0.272	0.257	0.800	0.016	0.098	0.081
	IRIV	52	0.760	0.065	0.256	0.240	0.789	0.007	0.089	0.062

**Table 4:** Comparison of results between CNN-Bi-LSTM model and Bayes-CNN-Bi-LSTM model.

Method	Calibration Set				Prediction Set			
	Rc^2^	RMSE	MSE	RPD	Rp^2^	RMSE	MSE	RPD
CNN-Bi-LSTM	0.960	0.042	0.002	5.349	0.889	0.074	0.005	3.131
Bayes-CNN-Bi-LSTM	0.932	0.059	0.004	4.073	0.909	0.067	0.004	3.445

## Data Availability

Data is contained within the article or Supplementary Material.

## Supplementary Materials

The following are available online at https://www.mdpi.com/article/10.3390/foods13030424/s1, Table S1: Statistical results of characteristic wavelength extraction. Table S2: Bayes-CNN-BI-LSTM model training parameters. Table S3: LSTM model training parameters.
