# Supplementary material for: Predictive Analysis of Linoleic Acid in Red Meat Employing Advanced Ensemble Models of Bayesian and CNN-Bi-LSTM Decision Layer Fusion Based Hyperspectral Imaging
Source: Foods. 2024 Jan 28;13(3):424. doi: 10.3390/foods13030424 (PMC10855435; doi:10.3390/foods13030424)
Supplement: Supplementary file 1 [file foods-13-00424-s001.zip › foods-2752200-supplementary.pdf]

Supplementary material

Table S1. Statistical results of characteristic wavelength extraction.

Method	Partition method	Number	Calibration set			Prediction set		
			Rc^2	RMSE	SE	Rp^2	RMSE	SE
PLSR	UVE	20	0.686	0.104	0.105	0.750	0.114	0.114
	VCPA	11	0.794	0.085	0.085	0.840	0.092	0.093
	CARS	17	0.792	0.085	0.086	0.820	0.094	0.095
	iVISSA	45	0.837	0.076	0.076	0.840	0.089	0.090
	IRIV	52	0.830	0.078	0.078	0.849	0.087	0.088

Table S2. Bayes-CNN-BI-LSTM model training parameters.

Hyperparameter name		Model
		Bayes-CNN-Bi-LSTM
CNN	L2 Regularization	0.001
	Kernel	1x3
	Dropout Layer	0.1
	Learn Rate Drop Factor	0.6
Bayes	Num Of Units	128
	Hidden layer	32
	Initial Learn Rate	0.05

Table S3. LSTM model training parameters.

Hyperparameter name	Model			
	LSTM	Bi-LSTM	CNN-Bi-LSTM	bayes-CNN-Bi-LSTM
**Units**	128	128	128	128
**Mini BatchSize**	16	16	32	32
**Max Epochs**	500	500	500	500
**Learningrate**	0.001	0.0009	0.01	0.01
**Learn Rate Drop Period**	100	100	50	50
**L2 Regularization**	0.001	0.001	0.0015	0.0015
